# Comparison of mNGS microbial detection profiles between percutaneous lung aspiration biopsy and bronchoalveolar lavage fluid in infective pneumonia

**DOI:** 10.1515/med-2026-1445

**Published:** 2026-06-01

**Authors:** Yinpeng Li, Guozhen Yi, Zhimei Han, Jianmei Fu, Leiqian Xu

**Affiliations:** Department of Respiratory and Critical Care, Hebei PetroChina Central Hospital, Langfang, China; Department of Stomatology, Hebei PetroChina Central Hospital, Langfang, China; Department of Surgery, Charite-University Medicine Berlin, Campus Benjamin Franklin (CBF), Berlin, Germany

**Keywords:** bronchoalveolar lavage, lung infection, metagenomic next-generation sequencing, microorganisms, percutaneous lung puncture biopsy

## Abstract

**Objectives:**

To compare the mNGS-based microbial detection profiles of percutaneous lung aspiration biopsy (PLAB) and bronchoalveolar lavage fluid (BALF) in patients with infective pneumonia under real-world clinical sampling strategies.

**Methods:**

The study included 166 patients with infective pneumonia, of whom 54 underwent PLAB to obtain unfixed fresh lung tissue from the lesion site, while 112 underwent fiberoptic bronchoscopy to obtain BALF.

**Results:**

In the BALF group, 3 pathogens of high concern and 5 suspected pathogens, totaling 8 types of pathogens, were detected. In contrast, in the PLAB group, 1 pathogen of high concern and 1 suspected pathogen, totaling 2 types of pathogens were detected. Cumulatively, 348 pathogens were identified in the BALF group. In the PLAB group, 96 pathogens were identified cumulatively, p<0.001. In the BALF group, the most frequently detected pathogen was *Streptococcus pneumoniae*, with 19 strains of *Mycobacterium tuberculosis* among the special pathogens. In the PLAB group, the most frequently detected pathogen was Epstein-Barr virus (EBV) (14.58 %).

**Conclusions:**

BALF and PLAB showed different mNGS microbial detection patterns under different clinical sampling strategies. Because of the retrospective non-paired design, these findings should be interpreted as descriptive comparative data rather than proof of the superior diagnostic performance of either sampling method.

## Introduction

Lung infections are the primary cause of pneumonia, with common pathogens including bacteria, viruses, fungi, chlamydia, mycoplasma, and parasitic worms. Transmission can occur through droplet and aerosol spread, aspiration or reflux of colonizing bacteria, weakened immune barriers, and nosocomial cross-infections, often exacerbated by underlying diseases. The diagnosis and treatment of pulmonary infections face significant challenges due to various factors such as changes in pathogens, drug resistance from irrational use of antibiotics, changes in population susceptibility, increased nosocomial pneumonia, and drug volume-based drug procurement. Accurate pathogen identification is crucial for etiological treatment. Historically, treatment has been empirical, based on patient epidemiology, clinical presentations, and imaging data. However, with advancements in diagnostic technology, metagenomic next-generation sequencing (mNGS) has gradually gained popularity alongside traditional sputum culture and microscopy [1], 2]. mNGS is particularly valuable for patients who do not respond to empirical antibacterial therapy, exhibit non-specific clinical symptoms, or are suspected of having specific bacterial infections [3]. This comprehensive analysis amplifies and sequences gene fragments, offering rapid, accurate, and broad-spectrum testing applicable to respiratory, central nervous system, and bloodstream infections. In pulmonary infections, particularly parenchymal infections in the form of pneumonia, sampling methods typically involve bronchoalveolar lavage or fresh tissue obtained from a CT-guided percutaneous lung biopsy. This study aimed to compare the mNGS microbial detection characteristics of these two clinically selected sampling strategies and to describe the potential strengths and limitations of each approach in real-world practice.

## Participants and methods

### Study participants

**Figure 1:**
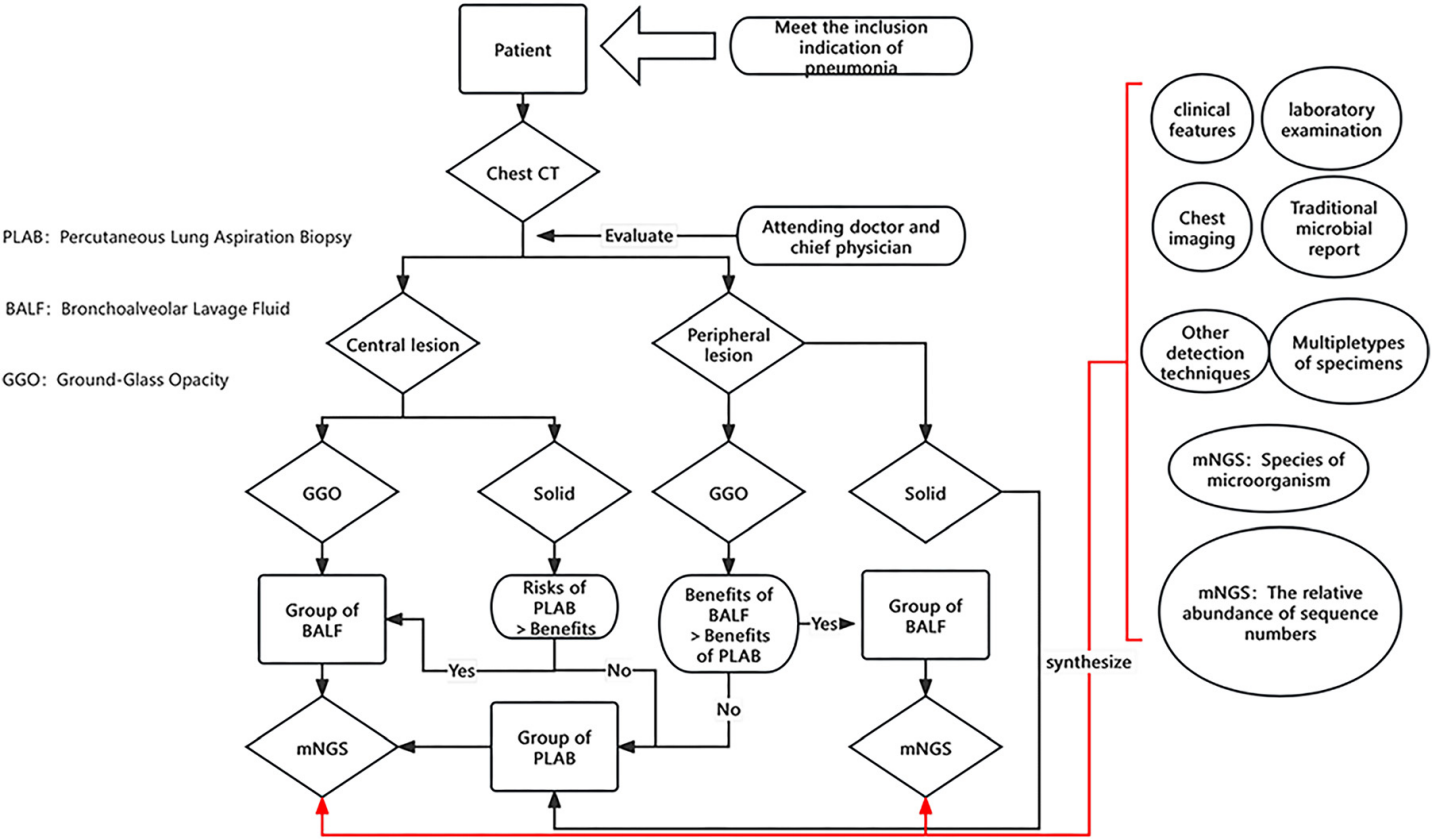
Flow chart.

Selection: From January 1, 2022, to September 1, 2022, a total of 166 patients diagnosed with infective pneumonia were admitted to the Department of Respiratory and Critical Care Medicine at Hebei PetroChina Central Hospital. The inclusion criteria for infective pneumonia were as follows: (1) causative factors, with clear clinical evidence of an infectious pneumonia caused by a pathogen; (2) new-onset clinical symptoms such as fever, cough, purulent sputum production, chest pain, dyspnea, and abnormal signs in lung examination such as consolidation and wet rales; (3) chest imaging indicating inflammatory changes; (4) blood routine showing elevated or decreased white blood cell count, abnormal C-reactive protein (CRP), procalcitonin (PCT), and other inflammatory markers. Exclusion criteria: (1) Cases involving pneumonitis due to non-infectious causes were excluded. (2) Since enrolled patients presented with clinical symptoms of infection upon admission requiring prompt treatment, the majority received empirical antibiotic therapy during hospitalization. However, to minimize potential confounding effects of antibiotic treatment on study outcomes, only patients who had undergone bronchoalveolar lavage or percutaneous lung biopsy within 48 h of antibiotic initiation were included in the final analysis. Chronic airway comorbidities such as COPD, asthma, and bronchiectasis were not prospectively collected as predefined study variables in this retrospective cohort. Therefore, a reliable subgroup analysis according to chronic airway disease status could not be performed. The choice of BALF or PLAB was not randomized but was made by the treating team according to lesion location, anatomical accessibility, procedural safety, and the patient’s overall clinical condition. Therefore, this study should be interpreted as a retrospective comparison of two clinically selected sampling strategies rather than a paired head-to-head diagnostic accuracy study. To improve comparability, all included patients were enrolled at the same institution during the same study period, met the same inclusion and exclusion criteria, and underwent mNGS testing using the same laboratory workflow. The details of the study participants are provided in Figure 1. Approval for this study was obtained from the Ethics Committee of the Petroleum Hospital. This study was conducted in accordance with the declaration of Helsinki. Written informed consent was obtained from all participants.

### Bronchoscopic bronchoalveolar lavage

After adequate local mucosal anesthesia (Local anaesthesia of the irrigated lung segment was administered by the injection of 1–2 mL of 2 % lidocaine through the biopsy port.), patients underwent bronchoscopy. The bronchial tubes at the lesion should be lavaged with 20 mL of 0.9 % sterile saline approximately 4–5 times. The irrigation fluid is then collected into a sterile plastic sampling bottle using a negative pressure suction device. It is imperative that the recovery rate is controlled to be more than 30 %. The specimens were collected in sterile containers with 10–20 mL of lavage fluid, labelled with specimen information and sent to the microbiology laboratory for testing within 2 h at room temperature. After operation, patients were instructed to abstain from food and water for 2 h to prevent aspiration pneumonia caused by choking and coughing.

### Percutaneous lung aspiration biopsy

The puncture site was marked using a marker pen under the guidance of a CT scan cursor and positioning ruler. A strictly aseptic procedure was maintained throughout the procedure. Local subcutaneous infiltration anesthesia was administered, and the syringe needle was positioned to determine the puncture angle and depth using a CT scan. As the needle was directed towards the target lesion, another CT scan was conducted to confirm the automated biopsy gun’s needle tip placement at the lesion’s edge or within it. The biopsy specimen intended for mNGS was placed in a sterile container without formalin and sent for sequencing analysis as unfixed fresh tissue. When histopathological examination was required, a separate core or the remaining tissue was fixed in 10 % neutral buffered formalin according to routine pathological practice. Patients were kept under observation for 2–4 h post-procedure to promptly manage pneumothorax and other complications. Following a biopsy, patients should be monitored for vital signs and oxygen saturation. In addition, a chest x-ray or chest CT should be completed within 24 h in order to monitor for complications such as pneumothorax, haemorrhage, air embolism, etc. Those exhibiting alterations in their condition should undergo a prompt review.

### mNGS and clinical interpretation

Bronchoalveolar lavage fluid and unfixed fresh tissue specimens were sent to Jiangsu Simcere Pharmaceutical Co. Ltd. for pathogen mNGS analysis. All samples were collected, preserved, and transported following relevant standards and biosafety protocols. In this study, only deoxyribonucleic acid (DNA) was sequenced, and ribonucleic acid (RNA) viruses were excluded from detection. Specimens were centrifuged, and DNA nucleic acid fragments were extracted using a quality control library and Illumina next-generation high-throughput sequencing technology. The process of quality control for sequencing data involves the removal of low-quality results and human reference gene sequences. This is achieved through the filtration of low-quality and low-complexity sequences, as well as the excision of splice sequences. The results were aligned against the dedicated microbial large database (SimcerePathogen-databaseV2.1) using advanced bioinformatics algorithms. Additionally, polymerase chain reaction (PCR) and repeated sequencing were conducted as needed to identify high-concern and suspected pathogens. The entire process underwent stringent multi-level internal and external quality control to ensure stable, reliable accuracy, and resistance to interference [4].

The diagnostic team comprised two senior physicians from the respiratory medicine department and two attending physicians from the radiology department. They identified relevant microorganisms by excluding false-positive pathogens and determining whether they were colonizing microorganisms. This was achieved through a comprehensive review of clinical symptoms, imaging features, serological test results, and mNGS reports. The interpretation of the original microbial species profile should be divided into two steps. Firstly, the confidence level of the detected microorganisms should be determined. This is to ascertain whether the microorganisms are actually present in the biological specimen. Secondly, the responsible pathogenic microorganisms that are clinically compatible with the detected microorganisms should be identified. These should be selected from among those that are detected with confidence. The following principles underpin the judgement criteria: 1. The higher the number of sequences or relative abundance of species of the same species, the higher the probability that the species is a pathogen (except in the bacterial part of the plant species). The analysis of bacteria, fungi, viruses, parasites, and special pathogens (such as Mycobacterium, Nucella, Pneumocystis japonicum, Mycoplasma, Chlamydia, Rickettsia, etc.) should be conducted separately. The number of sequences should take into account the genome size of different species (viruses<bacteria<fungi<parasites) and the ease of nucleic acid extraction efficiency (viruses<Gram-negative bacteria<Gram-positive bacteria<fungi<*Mycobacterium tuberculosis*). 2. Parasite gene sequencing results should always be checked to see if the comparison sequences are the only comparison sequences as well as the genome coverage, in order to reduce the rate of false positives. 3. In the event of the presence of colonising microorganisms in the specimen, the results should be analysed. In the event of the presence of colonising microorganisms in the specimen, the relative abundance of each type of microorganism should be sorted in descending order, and analysed and judged one by one in conjunction with the pathogenicity of known pathogenic microorganisms. It is also noteworthy that colonising microorganisms in specimens from special patients, such as those with trauma, tumours, or who are immunosuppressed, may also be the responsible pathogenic microorganisms. In routine clinical practice, conventional microbiological data, including bacterial culture, PCR, serological tests, imaging findings, and subsequent treatment response, were considered as part of the multidisciplinary interpretation of mNGS results whenever available. However, the present study was not designed as a formal concordance analysis between mNGS and conventional microbiological methods. Case-level one-to-one matching between mNGS results and contemporaneous culture results was not prospectively predefined or systematically recorded for all patients, and therefore a reliable comparative analysis of correspondence between PLAB/BALF mNGS findings and bacterial culture could not be performed in the current dataset. For analytical purposes, microorganisms reported by mNGS were categorized into three levels: total detected microorganisms, pathogens requiring high attention, and suspected pathogens. Total detected microorganisms referred to all taxa passing laboratory quality control and bioinformatic filtering. Pathogens requiring high attention referred to microorganisms with high technical confidence and strong etiological plausibility for pneumonia, whereas suspected pathogens referred to microorganisms that were technically credible but still required multidisciplinary clinical adjudication because colonization, upper-airway carryover, latent/reactivated virus, or mixed infection could not be excluded. Categorization was based on specimen type, sequencing reads, relative abundance, genomic coverage, known pathogenicity, host status, imaging findings, and corroborative evidence from PCR, culture, serology, or treatment response when available. A study-specific list of representative microorganisms assigned to each category is provided in Supplementary Table S1.

### Statistical analysis

Statistical analyses were performed using SPSS version 26.0 to evaluate the significance of differences in various markers across different specimen acquisition groups. Qualitative data were expressed as percentages, and the significance of differences was assessed using the chi-squared test and Mann Whitney-U test. A p-value<0.05 was considered statistically significant.

### Ethics approval and consent to participate

Approval for this study was obtained from the Ethics Committee of the Petroleum Hospital. This study was conducted in accordance with the declaration of Helsinki. Written informed consent was obtained from all participants.

## Results

### Total pathogen categories

A total of 166 patients were included in the study, comprising 82 females (49.40 %) and 84 males (50.60 %). There were 112 patients in the bronchoscopic alveolar lavage (BALF) group, of which 58 were females (51.79 %) and 54 were males (48.21 %). During the study, no subjects exhibited symptoms of bloody lavage fluid due to mucosal bleeding from excessive negative pressure. Furthermore, no subjects demonstrated a significant decrease in oxygen saturation as a result of the saline injections. The recovery of lavage fluid was greater than 30 % for all subjects. There were 54 patients in the percutaneous lung aspiration biopsy (PLAB) group, of which 24 were females (44.44 %) and 30 were males (55.56 %). The chi-squared test showed a p value of 0.714>0.05, indicating no statistically significant difference in sex distribution between the groups. In the cohort of 54 patients who underwent lung puncture biopsy, one patient was found to have pneumothorax (1.85 %), while six patients exhibited self-limited small haemoptysis or blood in the sputum (11.1 %). It is noteworthy that no patient demonstrated a pleural reaction during the procedure, and no serious complication, such as air embolism, was observed. The mean age was 58.84 years in the BALF group and 61.76 years in the PLAB group, with a p value equal to 0.599>0.05, indicating that the age difference was not statistically significant (see Table 1).

**Table 1:** Comparison of pathogen species detected by two sampling methods.

Variable	Total (n=166)	Bronchoalveolar lavage fluid (n=112)	Lung puncture (n=54)	Statistic	p-Value
Sex, n (%)				1.064	0.714
Female	82 (49.40)	58 (51.79)	24 (44.44)		
Male	84 (50.60)	54 (48.21)	30 (55.56)		
Suspected pathogens, median [IQR]	4.00 [1.00, 6.00]	5.000 [3.00, 7.00]	1.000 [1.00, 2.00]	8.207	<0.001
Pathogens of high concern, median [IQR]	2.00 [1.00, 4.00]	3.000 [2.00, 4.00]	1.000 [0.00, 2.00]	6.396	<0.001
Total pathogens, median [IQR]	6.00 [3.00, 9.00]	8.00 [6.00, 11.000]	2.000 [1.00, 4.00]	8.864	<0.001
Age, mean (±SD)	59.79 ± 15.62	58.84 ± 17.33	61.76 ± 11.04	−0.527	0.599

**Figure 2:**
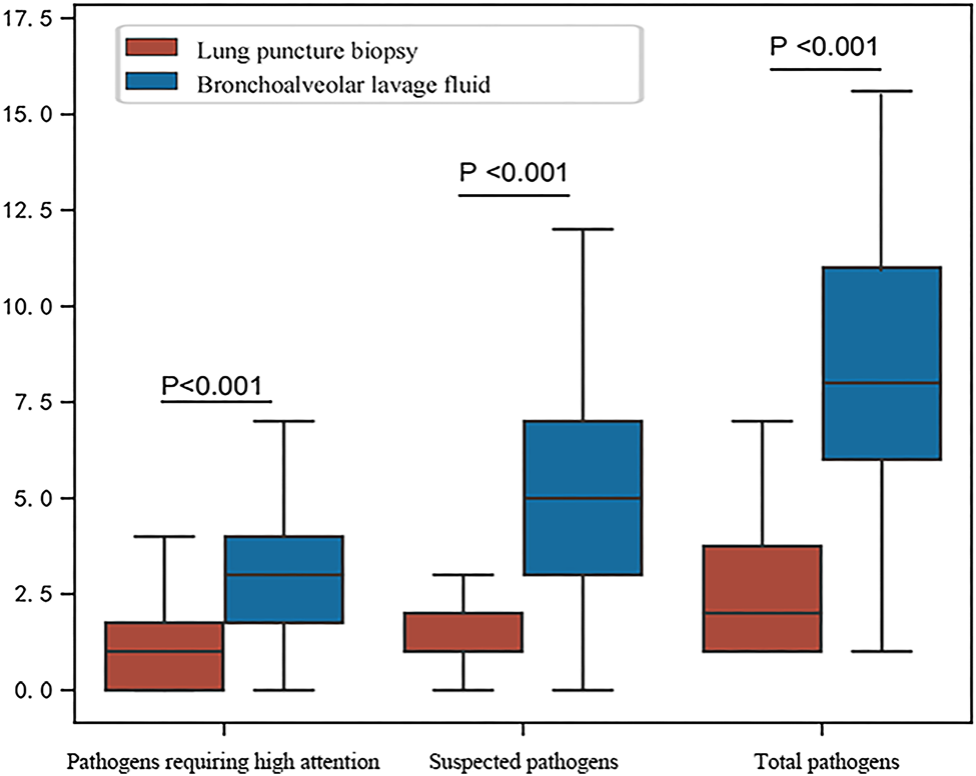
Boxplots of the number of microorganisms detected per patient by sampling method. The y-axis shows microorganism counts per patient.

For pathogens of high concern, an average of 3 microorganisms were detected in the BALF group compared to 1 in the PLAB group. For suspected pathogens, a total of 5 microorganisms were detected on average in the BALF group, while 1 was detected in the PLAB group. For total pathogens, a total of 8 microorganisms were detected on average in the BALF group, while 2 were detected in the PLAB group. The Mann Whitney-U test was used for intergroup comparison, and all p values were <0.001. Hence, the differences between the groups were statistically significant (see Figure 2).

### Detection rates of different pathogens

A total of 348 pathogens were detected in the BALF group, of which 159 were Gram-positive bacteria (45.7 %), 114 were Gram-negative bacteria (32.8 %), 41 were viruses (11.8 %), 23 were *M. tuberculosis* (6.6 %), and 11 were fungi (3.2 %). In contrast, 96 pathogens were detected in the PLAB group, of which 26 g-positive bacteria (27.1 %), 29 g-negative bacteria (30.2 %), 24 viruses (25 %), 10 fungi (10.4 %), and 7 tuberculosis (7.3 %). The Mann Whitney-U test showed a p value of <0.001 between the above groups, indicating statistically significant differences between the groups (see Table 2).

**Table 2:** Comparison of the detection rates of the two sampling methods for different pathogens.

Variable	Total	Bronchoalveolar lavage fluid	Lung puncture	Statistic	p-Value
Bacteria					
Gram-positive	^a^185 (41.7)	^a^159 (45.7)	^a^26 (27.1)		
Gram negative	^a^143 (32.2)	^a^114 (32.8)	^a^29 (30.2)		
Fungi	^a^21 (4.7)	^a^11 (3.2)	^a^10 (10.4)	23.81	<0.001
Virus	^a^65 (14.6)	^a^41 (11.8)	^a^24 (25.0)		
Tuberculosis	^a^30 (6.8)	^a^23 (6.6)	^a^7 (7.3)		
Total	444	348	96		

^a^Results are cumulative for pathogen species for all patients in each group. %, number of detections/total.

### Distribution of major pathogens

In the BALF group, the most frequently detected pathogens were *Streptococcus pneumoniae* (64 strains, accounting for 18.39 %), followed by *Haemophilus parainfluenzae* (30 strains, accounting for 8.62 %), and *Streptococcus pseudopneumoniae* (25 strains, accounting for 7.18 %). Among fungi, *Candida albicans* was the most abundant (20 strains, accounting for 5.75 %). Among special pathogens, 19 strains of *M. tuberculosis* were detected, accounting for 5.46 %, and among viruses, the herpes virus was the most abundant (10 strains, accounting for 2.87 %). In the PLAB group,the most detected pathogens were EBV (14 strains, accounting for 14.58 %), followed by *M. tuberculosis* (7 strains, accounting for 7.29 %) and *Klebsiella pneumoniae* (5 strains, accounting for 5.21 %). Four strains were detected each for *Staphylococcus aureus*, cytomegalovirus, *Staphylococcus maltophilia*, and *S. pneumoniae* (accounting for 4.17 %) (see Table 3).

**Table 3:** Distribution of the main pathogens detected by the two sampling methods.

Variable	Bronchoalveolar lavage fluid	Variable	Lung puncture
Major pathogens		Major pathogens	
*Streptococcus pneumoniae*	64 (18.39)	EBV	14 (14.58)
*Haemophilus parainfluenzae*	30 (8.62)	*Mycobacterium tuberculosis*	29 (30.21)
*Streptococcus pneumoniae*	25 (7.18)	*Klebsiella pneumoniae*	5 (5.21)
*Candida albicans*	20 (5.75)	*Staphylococcus aureus*	4 (4.17)
*Mycobacterium tuberculosis*	19 (5.46)	Cytomegalovirus	4 (4.17)
*Haemophilus influenzae*	16 (4.60)	*Stenotrophomonas maltophilia*	4 (4.17)
*Staphylococcus aureus*	15 (4.31)	*Streptococcus pneumoniae*	4 (4.17)
*Klebsiella pneumoniae*	14 (4.02)	*Enterococcus faecalis*	3 (3.13)
*Pseudomonas aeruginosa*	14 (4.02)	*Pseudomonas aeruginosa*	3 (3.13)
Herpes virus	10 (2.87)		

The variables “pathogens requiring high attention” and “suspected pathogens” were used as clinically adjudicated analytical categories rather than binary endpoints; therefore, the present study did not further calculate their patient-level positive rates as a surrogate of diagnostic sensitivity.

## Discussion and conclusion

In this study, the average number of suspected pathogen types, the species of pathogens requiring high concern, and the total category of total pathogens detected per sample in the PLAB group were lower than those in the BALF group. This suggests that PLAB can reduce the interference of contaminating bacteria, thereby minimizing the misleading effects of background or colonizing bacteria on clinical interpretation and judgment. Consequently, PLAB enhances the microbiological diagnostic value of mNGS. However, due to the small sample size of the PLAB group, expanding the sample size may be necessary to exclude the false-negative rate associated with lung puncture. Bacteria remained the predominant pathogen in both the BALF and PLAB groups, with the BALF group showing a predominance of Gram-positive and the PLAB group showing the opposite result. This result may be due to interference by respiratory colonizing bacteria, attributable to the fact that the PLAB group is capable of effectively reducing the presence of oral colonisation bacteria in the results by employing good aseptic puncture technique, highlighting the advantage of the PLAB group in avoiding the influence of background bacteria. Additionally, the PLAB group demonstrated superior detection rates of specific pathogenic bacteria compared to the BALF group, suggesting that it may be more effective in detecting *M. tuberculosis*, fungi, and viruses. Therefore, there is a basis to consider that the percutaneous lung puncture technique may be more effective than bronchoscopy for mNGS testing.

Compared with traditional tests such as staining, microscopic examination, and culture, mNGS demonstrates unique properties in diagnosing lung infections. Qin’s study highlights its effectiveness in the detection of specific pathogens such as viruses, fungi, and *M. tuberculosis* over conventional methods [5]. Similarly, Jin reported a high positive predictive value of 97.46 % using mNGS on bronchoalveolar lavage fluid from 246 patients with suspected lung infections [6]. This new method partly addresses the issues of lengthy turnaround times and false positives associated with sputum or blood cultures [7]. mNGS is suitable for a broad range of specimens, including sputum, bronchoalveolar lavage fluid, pharyngeal swabs from the respiratory tract, fresh tissue, and blood samples. Several studies have demonstrated its significant role in diagnosing and treating pulmonary infections, particularly in severe or unexplained cases [8], 9]. The sensitivity of mNGS depends on sample cell density and microbial concentration, with potential limitations in specimens where pathogen content in the is below detection thresholds due to sample collection methods or prior antibiotic use, leading to false negatives. Therefore, integrating mNGS with low-cost traditional detection techniques is recommended to accurately identify causative agents [10].

Although mNGS technology and its databases are constantly evolving and improving, the complexity of its procedures can still lead to environmental microorganisms contaminating samples, resulting in false positives [11]. These contaminants often originate from the sample collection and transfer process, as well as from sampling personnel. Additionally, the rich diversity of human microbiome flora can contribute to false positive results [12]. This effect arises from colonizing microorganisms found in multiple patient sites, including the oral and nasal cavities, gastrointestinal tract, and reproductive tract [13].

Bronchoalveolar lavage is a low-risk diagnostic operation designed to retrieve infectious agents from the distal respiratory tract and alveolar cavity, characterized by minimal injury and a lower complication rate of approximately 0–2.3 % compared to percutaneous lung puncture biopsy. The most common complication is a transient postoperative fever. In addition, the lavage technique removes airway secretions and achieves certain therapeutic effects in patients with lung infections [14]. A CT-guided percutaneous lung aspiration biopsy is primarily used to obtain fresh lesion tissue precisely, which is more suitable for lesions close to the lung periphery. This method requires the synergistic cooperation of imaging departments, as tissue necrotic lesion specimens have limited diagnostic value, and the experience and skill level of the surgeon directly determines the validity of the sample. [15]. It also reduces nasopharyngeal discomfort, coughing, and reflux during the procedure. Although alveolar lavage is less likely to miss the site of infection when sampling after multiple lung segment lavages, BAL does not consistently align with the sampling site and pathogenic organism results as effectively as lung puncture. CT-guided systems can be used to localize biopsies at sites with prominent imaging abnormalities, yielding higher diagnostic rates and minimizing interference from background organisms [16]. However, due to the puncture needle passing directly through the pleura and lung tissue, multiple biopsies may be necessary to increase sensitivity. Puncture sites adjacent to the heart or large blood vessels pose an increased risk of hemorrhage and pneumothorax, which limits routine use for diagnosing lung infections [17].

Infectious diseases are primarily caused by living pathogens, and their evolution within the human body differs fundamentally from diseases caused by other factors. Lung infections can encompass both infectious and non-infectious origins, and infectious pneumonia caused by pathogens accounts for a large proportion of these, exemplified by global events such as the coronavirus disease 2019 (COVID-19) pandemic, which has resulted in significant losses to lives, property, and national economies. Hence, rapid and accurate diagnosis of the causative agent is crucial for personalized diagnosis and treatment of pneumonia.

This study has several limitations. This study employed a retrospective, non-paired design, with BALF or PLAB selected based on individualized clinical needs, potentially introducing selection bias. Consequently, our results are more appropriately interpreted as a comparison of mNGS microbial detection characteristics under different clinical sampling strategies, rather than as proof of strict superiority in diagnostic performance between the two methods.

Clinical response after treatment may provide supportive retrospective information for assessing the plausibility of pathogen interpretation, but treatment-related outcomes were not predefined study endpoints in the present analysis and therefore cannot be used to establish an independent therapeutic benefit of either sampling strategy. Because chronic airway disease status was not uniformly documented using predefined criteria, the present study could not reliably assess the influence of COPD, asthma, or bronchiectasis on mNGS interpretation. Future prospective studies should systematically record such comorbidities and evaluate their impact on BALF- and PLAB-based microbial detection profiles.

## Conclusions

In conclusion, although new diagnostic techniques can significantly improve the detection rate and sensitivity of pulmonary infection pathogens, complementary methods remain extremely important in clinical practice. Understanding the strengths and limitations of each technique, timely combining their applications, and developing personalized sampling and diagnostic protocols based on the patient’s lesion and physical condition can mitigate risks and optimize outcomes. The rational choice of bronchoscopy or percutaneous lung puncture techniques maximises the effectiveness of mNGS testing and yields the most reliable diagnostic results.

## Supplementary Material

Supplementary Material

## References

[1] Han D, Li Z, Li R, Tan P, Zhang R, Li J (2019). mNGS in clinical microbiology laboratories: on the road to maturity. Crit Rev Microbiol.

[2] Jiang XW, Liang ZK, Zeng L, Yuan YL (2023). Results analysis of mNGS applied to infectious diseases. Zhonghua Yufang Yixue Zazhi.

[3] Xie G, Zhao B, Wang X, Bao L, Xu Y, Ren X (2021). Exploring the clinical utility of metagenomic next-generation sequencing in the diagnosis of pulmonary infection. Infect Dis Ther.

[4] Chinese Pharmacists Association; Branch of Bacterial Infection and Resisatnce Prevention of Chinese Medical Association; Expert Committee of the National Health Commission on Antimicrobial Susceptibility Testing and Standard Research (2024). Expert consensus on clinical localization detection standards for metagenomic next generation sequencing of pathogens. Zhonghua Yufang Yixue Zazhi.

[5] Qin Z, Zou Y, Huang Z, Yu N, Deng Z, Chen Z (2022). Metagenomic next-generation sequencing contributes to the diagnosis of mixed pulmonary infection: a case report. Ann Clin Microbiol Antimicrob.

[6] Jin X, Li J, Shao M, Lv X, Ji N, Zhu Y (2022). Improving suspected pulmonary infection diagnosis by bronchoalveolar lavage fluid metagenomic next-generation sequencing: a multicenter retrospective study. Microbiol Spectr.

[7] Yang L, Song J, Wang Y, Feng J (2021). Metagenomic next-generation sequencing for pulmonary fungal infection diagnosis: Lung biopsy versus bronchoalveolar lavage fluid. Infect Drug Resist.

[8] Li N, Ma X, Zhou J, Deng J, Gu C, Fei C (2022). Clinical application of metagenomic next-generation sequencing technology in the diagnosis and treatment of pulmonary infection pathogens: a prospective single-center study of 138 patients. J Clin Lab Anal.

[9] Qian YY, Wang HY, Zhou Y, Zhang HC, Zhu YM, Zhou X (2021). Improving pulmonary infection diagnosis with metagenomic next generation sequencing. Front Cell Infect Microbiol.

[10] Wang J, Han Y, Feng J (2019). Metagenomic next-generation sequencing for mixed pulmonary infection diagnosis. BMC Pulm Med.

[11] Chiu CY, Miller SA (2019). Clinical metagenomics. Nat Rev Genet.

[12] Pan X, Zhang Y, Chen G (2023). The clinical utility of metagenomic next-generation sequencing for the diagnosis of central nervous system infectious diseases. Neurol Res.

[13] Morsli M, Salipante F, Magnan C, Dunyach-Remy C, Sotto A, Lavigne JP (2024). Direct metagenomics investigation of non-surgical hard-to-heal wounds: a review. Ann Clin Microbiol Antimicrob.

[14] Hogea SP, Tudorache E, Pescaru C, Marc M, Oancea C (2020). Bronchoalveolar lavage: role in the evaluation of pulmonary interstitial disease. Expert Rev Respir Med.

[15] Chan MV, Afraz Z, Huo YR, Kandel S, Rogalla P (2023). Manual aspiration of a pneumothorax after CT-guided lung biopsy: outcomes and risk factors. Br J Radiol.

[16] Nath A, Neyaz Z, Hashim Z, Agrawal V, Richa M (2019). Role of percutaneous computed tomography-guided lung biopsy in non-resolving consolidation and identification of clinical and high-resolution computed tomography characteristics predicting outcome. J Clin Imaging Sci.

[17] Polat G, Özdemir Ö, Serçe Unat D, Karadeniz G, Ayrancı A, Unat ÖS (2023). Pneumothoraxes after CT-guided percutaneous transthoracic needle aspiration biopsy of the lung: a single-center experience with 3426 patients. Akciğere BT kılavuzluğunda perkütan transtorasik iğne aspirasyon biyopsisi sonrası pnömotorakslar: 3426 hasta ile tek merkez deneyimi. Tuberk Toraks.

