# Supplementary material for: Comparison of mNGS microbial detection profiles between percutaneous lung aspiration biopsy and bronchoalveolar lavage fluid in infective pneumonia
Source: Open Med (Wars). 2026 Jun 1;21(1):20261445. doi: 10.1515/med-2026-1445 (PMC13225183; doi:10.1515/med-2026-1445)
Supplement: Supplementary file 1 — Supplementary Material [file j_med-2026-1445_suppl_001.docx]

**Supplementary Table S1. Study-specific operational categorization of microorganisms detected by mNGS in this cohort**

| **Category** | **Operational definition used in this study** | **Representative microorganisms/examples** | **Interpretation notes** |
| --- | --- | --- | --- |
| **Pathogens requiring high attention** | Microorganisms with high technical confidence and strong etiological plausibility for pneumonia; relatively unlikely to represent routine airway colonization or background contamination in the given specimen type; detection concordant with clinical presentation and radiological findings, with corroborative evidence when available. | Mycobacterium tuberculosis complex; Nocardia spp.; Pneumocystis jirovecii; Legionella spp.; Mycoplasma pneumoniae; Chlamydia psittaci; Chlamydia pneumoniae; Aspergillus spp. (when supported by host factors and imaging); Cryptococcus neoformans/gattii; Mucorales. | This category was used for microorganisms that usually require immediate clinical attention because of recognized pulmonary pathogenicity and potential treatment implications. |
| **Suspected pathogens** | Microorganisms detected with technical confidence and possible relevance to the current infection, but still requiring multidisciplinary adjudication because colonization, upper-airway carryover, latent/reactivated virus, or mixed infection could not be excluded. | Streptococcus pneumoniae; Haemophilus influenzae; Haemophilus parainfluenzae; Klebsiella pneumoniae; Staphylococcus aureus; Pseudomonas aeruginosa; Acinetobacter baumannii; Stenotrophomonas maltophilia; aspiration-associated anaerobes; Epstein-Barr virus (EBV); Cytomegalovirus (CMV); other herpes viruses. | This category was used when the organism was plausible but not definitively etiological on the basis of mNGS alone. Viral organisms such as EBV/CMV/herpes viruses were interpreted cautiously and were not automatically considered causative pathogens. |
| **Total detected microorganisms** | All microorganisms passing laboratory quality control and bioinformatic filtering and included in the final report, regardless of final clinical interpretation. | All reported bacteria, fungi, viruses, parasites, and special pathogens. | This category was used for descriptive counting only and should not be interpreted as equivalent to etiological pathogens. |
| **Background/colonizing microorganisms interpreted cautiously*** | Organisms commonly encountered as oral or airway background flora or colonizers in respiratory specimens, unless strong clinical evidence supported pathogenicity. | Oral streptococci (including mitis-group streptococci); Neisseria spp.; Rothia spp.; Prevotella spp.; Veillonella spp.; Gemella spp.; Actinomyces spp.; Candida spp. in respiratory specimens. | These organisms were not automatically classified as pathogens requiring high attention. Their clinical significance depended on specimen type, abundance, host factors, and concordance with imaging and clinical findings. |

**Note: This row is provided to improve transparency of interpretation; these organisms were not a formal statistical category in the primary analyses.*
